# "Really Heroic Work": The Unequalled Efficiency of the R.A.M.C.

**Published:** 1914-11-28

**Authors:** 


### SURGICAL EXPERIENCES IN THE PRESENT WAR.

### "REALLY HEROIC WORK."

### The Unequalled Efficiency of the R.A.M.C.

The resumed debate on this subject was held at the rooms of the Medical Society of London on Monday, 23rd inst., under the presidency of Sir John Bland Sutton.

### Mr. D'ARCY POWER

said the wounds which had come under his observation as results of the war had been caused by shrapnel casing, shrapnel bullets —of which there were at least two varieties, one well finished and in a nickel envelope, the other a mere rough leaden bullet—conical bullets, and large shell. He had had to deal with no lance, bayonet, sword, or sabre wounds. The latter cases had probably not got beyond the field ambulances. He showed two sovereigns which had been driven into the thickest part of the erector spinæ of an officer who was wearing a money-belt. One coin bore the impression of the other, even to its milled edge. Though driven into the body together, they lay widely apart in the muscle. One of the coins had comminuted the transverse process of one of the vertebræ and torn away part of the crest of the ilium. He had had many cases of spinal injury, and some of concussion of the cord and peripheral nerves, as there was no obvious lesion. He had been struck by the number of complete perforations, in which only the points of entrance and exit had suppurated, the intervening track being completely healed before the patients reached the base hospital. Where sinuses existed, he had not hesitated, where possible, to lay them freely open. It was only by complete simplification of a lacerated wound that its thorough cleansing was possible. His principles of treatment had been free opening, compresses, fomentations, or constant irrigation. He did not favour dry dressings, as they adhered to the wound. Many of the wounds had sloughed more than they had suppurated, and in some cases amputation had been necessary. Before an operation was undertaken in the case of sloughing wounds, it had been their custom to have a bacteriological examination made of the secretion, especially with the view of determining whether tetanus bacilli were present. If these were found, a prophylactic dose of anti-tetanic serum was administered the day prior to operation. He preferred injection of it by lumbar puncture. He believed that at present surgeons were too meddlesome in the case of wounds of the chest. In many such cases, even though the temperature be high, it seemed only necessary to aspirate as often as needed. He did not extract all bullets simply because skiagrams showed them *in situ*. In nearly all cases in which wounds had become painful after healing exploration was rewarded by the discovery of a foreign body. It should never be forgotten that the tissues of a healthy man showed a marvellous power of repair.

### Mr. CHARTERS SYMONDS

thought much good must result from the interchange of opinions, for all surgeons did not treat these injuries in the same way. He dealt chiefly with the management of septic comminuted fractures and of wounds to joints, for in these cases both life and limb were in peril, and the surgeon's effort in the first place should be concentrated upon the saving of both, leaving refinements to a later period. The cases he had had to deal with arrived on the second to the tenth day after the infliction of the injury. Even in forty-eight hours they were septic, and later the wounds were so offensive as to be almost unbearable. Yet the patients' general condition was usually good. Sometimes the wounded man lay on the field two or even three days before receiving any attention. But there had been no lack of attention and skilful dressing of the patient on his way home, and he paid a tribute of praise not only to the medical officers, but also to the Red Cross nurses. The constitutional disturbance in these cases from the war was much less than in the cases of wounds met with in civil practice. He narrated several cases which confirmed Sir Watson Cheyne's contention that stronger limbs often resulted from simple treatment and but little interference, even though there might be some imperfections of alignment. He concluded by dealing with secondary hæmorrhage, methods of treatment of cases which were critically ill, and the kind of injury which needed the application of a plate.

### Mr. R. P. ROWLANDS

emphasised the necessarily dirty condition of the wounded patients, and the unavoidable delay in treatment. The latter he regarded as the crux of the whole matter. It was almost impossible to apply Sir Watson Cheyne's advice in the conditions met with in the present war, for in very many cases no cleansing could be done within twenty-four hours of the receipt of the injury. Their gallant *confrères* in France were doing really heroic work. He pleaded for the appointment of a committee, consisting of surgeons, bacteriologists, and chemists, to discuss the varieties, merits, and suitable strengths of different antiseptics. For instance, his colleague, Mr. Philip Turner, found that a 1 per cent. solution of picric acid in methylated spirit was more efficient than an ordinary iodine solution, and only about one-tenth of the cost. He did not find iodine was a sufficient cleanser of lacerated muscles. Radical operations for the repair of nerves, tendons, and bones should be deferred until sepsis had subsided. He attached very great importance to efforts to get the wounded quicker to the field or base hospitals, especially by motor field ambulances. There had been very great congestion at the field hospitals, yet there was no real lack of medical men, and many willing to co-operate had not been asked. Bad cases should be moved as little as possible. Many cases of stiff joints would have been avoided if the patient had been rested in the field hospital, for much travelling resulted in a joint being filled with pus. The R.A.M.C. service had been brought to a state of efficiency unequalled by that of any other army, but much yet remained to be done for the greater comfort and the better treatment of the wounded.

**Dr. IRONSIDE BRUCE**

gave a very instructive demonstration of skiagrams of various types of injury, and proceeded to discuss the localisation of foreign bodies by means of $x$-rays. The mere statement that a foreign body was at a certain depth beneath the surface conveyed nothing anatomically, because muscle and tissue varied so much in thickness in various people. To use localisation for extracting a foreign body it was necessary to make a vertical plane, but that could not always be followed at operations, and then the method became useless.

**Mr. W. H. JESSOP**

spoke of four cases which had come over from the war absolutely blind, yet after eight or nine days in hospital sight had been completely restored, and ophthalmoscopically there was nothing abnormal to be seen. There were also two cases of blepharospasm in apparently blind Belgians. They recovered in a few days. He believed those results had ensued from very heavy shell fire, to which all the cases had been exposed. Cases of septic iritis had also been seen, due to the gonococcus.

**Mr. JOCELYN SWAN,**

speaking from an experience of 2,000 cases since the war began, agreed with Sir Watson Cheyne that these cases must receive antiseptic treatment, and that treatment only, for a large proportion of the cases were septic. A very important thing was to secure and maintain adequate drainage. It was essential to decide what was the best form of treatment at the Front, as distinct from that to be carried out at the base. All whom he had spoken to on the subject agreed as to the great difficulty in getting at the patients for their first treatment; despite the fact that many of the doctors were working under fire. Most of the wounded had to be rescued in the darkness. When wounds were septic, he did not hesitate to open them up freely, washing them out with peroxide, weak carbolic, or weak permanganate, and applying a drain at the most dependent parts. Most of the infections were mixed—staphylococcus, streptococcus and coliform bacilli of the Proteus group. He had had ten cases of tetanus under his personal care, of which two succumbed, and these had had a limb amputated, When tetanic symptoms developed, he thought amputation should be delayed. Three of his cases from the Front had malignant œdema. He had had eighteen cases in which bullets had entered the brain; in some they had passed right through. Two of them got well after relieving the depressed fracture which occurred at the point of entry. Sometimes there was a very small scalp wound, but an extensive fracture underlying it. Some gutter fractures caused much comminution of the inner table. Skull injuries should be tackled straight away. In the case of thoracic and abdominal injuries, he did not interfere unless symptoms demanded it.

**THE PRESIDENT**

agreed with Sir Watson Cheyne's opening remarks, but said that if surgeons simply followed in the paths made by their predecessors, there would be no advance, and the paths would become ruts. Surgeons whose minds were sufficiently plastic to adopt the best in antisepsis and asepsis need have little anxiety as to results. He preferred iodine as an antiseptic. The initial sepsis in the cases from the Front he believed to be largely due to the conditions by which the soldiers were surrounded. Even in civil practice, with good surgeons and keen house surgeons, sepsis occasionally attacked wounds.

